# Patterns of individual compliance with anthelmintic treatment for soil-transmitted helminth infections in southern Ethiopia over six rounds of community-wide mass drug administration

**DOI:** 10.1093/trstmh/trad079

**Published:** 2023-11-15

**Authors:** R Maddren, B Collyer, A E Phillips, S Rayment Gomez, B Abtew, U Anjulo, D Tadele, A Sharma, A Tamiru, E Firdawek Liyew, M Chernet, R M Anderson

**Affiliations:** Imperial College London, St Mary's Campus, Praed Street, London W2 1NY, UK; Imperial College London, St Mary's Campus, Praed Street, London W2 1NY, UK; FHI360, 359 Blackwell Street, Suite 200, Durham, NC, USA; Imperial College London, St Mary's Campus, Praed Street, London W2 1NY, UK; Imperial College London, St Mary's Campus, Praed Street, London W2 1NY, UK; Federal Ministry of Health, 1234 Sudan Street, Addis Ababa, Ethiopia; Simprints, Cambridge CB1 2FH, UK; Simprints, Cambridge CB1 2FH, UK; Federal Ministry of Health, 1234 Sudan Street, Addis Ababa, Ethiopia; Ethiopian Public Health Institute, Swaziland Street, 2PWJ P8C, Addis Ababa, Ethiopia; Ethiopian Public Health Institute, Swaziland Street, 2PWJ P8C, Addis Ababa, Ethiopia; Imperial College London, St Mary's Campus, Praed Street, London W2 1NY, UK

**Keywords:** control program, individual longitudinal compliance, mass drug administration, neglected tropical diseases, never treated, soil-transmitted helminths, transmission break

## Abstract

**Background:**

The mainstay of soil-transmitted helminth (STH) control is repeated mass drug administration (MDA) of anthelmintics to endemic populations. Individual longitudinal compliance treatment patterns are important for identifying pockets of infected individuals who remain untreated and serve as infection reservoirs.

**Methods:**

The Geshiyaro Project censused the study population in Wolaita, Ethiopia at baseline in 2018. Individual longitudinal compliance was recorded for six rounds of community-wide MDA (cMDA). The probability distribution of treatment frequency was analysed by age and gender stratifications. Probabilities of transmission interruption for different compliance patterns were calculated using an individual-based stochastic model of *Ascaris lumbricoides* transmission.

**Results:**

The never-treated (0.42%) population was smaller than expected from a random positive binomial distribution. The observed compliance frequency was well described by the beta-binomial distribution. Preschool-age children (odds ratio [OR] 10.1 [95% confidence interval {CI} 6.63 to 15.4]) had the highest never-treated proportion of the age groups. Conversely, school-age children (SAC) and adults (OR 1.03 [95% CI 0.98 to 1.09]) had the highest always-treated proportion of the age groups.

**Conclusions:**

The study reports the largest dataset of individual longitudinal compliance to cMDA for STH control. Clear pattens are shown in the age-dependent distribution of individual compliance behaviour. The impact of compliance on the probability of elimination is significant, highlighting the importance of recording the full frequency distribution, not just the never-treated proportion.

## Introduction

Mass drug administration (MDA) is the predominant intervention employed to control human helminth infections, including the soil-transmitted helminths (STHs).^1^ These parasites do not induce strong acquired immunity and therefore repeated treatment is required to reduce morbidity and transmission. This is deployed at time intervals dictated by the target parasite's transmission dynamics to the most susceptible age groups in a given endemic locality. The global adoption of MDA programs has been enabled by drug donations from pharmaceutical companies, the delivery of which is financially supported by philanthropic organisations. As a result, the number of communities globally with high prevalence of helminth infections has decreased markedly over the past decades, as recently documented in the World Health Organization (WHO) 2030 roadmap progress report.^2^ Control impact is due to both sustained MDA efforts (including the beneficial overlap of programs in areas co-endemic with lymphatic filariasis) and improved water, sanitation and hygiene (WASH) infrastructure, often linked to socio-economic development or the result of government and philanthropic investment.^3^ Consequent to this success, national programs are increasingly evolving away from morbidity control and toward elimination as a public health problem, and ultimately the interruption of transmission.

MDA is highly efficient in significantly reducing a large proportion of community infection. However, transitioning from low prevalence to elimination as a public health problem, and finally to elimination of transmission, requires increased programmatic coverage of MDA and WASH interventions uniformly across the remaining endemic regions. High-quality monitoring and evaluation of MDA programs is therefore essential to record program impact and subsequent identification of target areas requiring improvement. This is well demonstrated in the Expanded Special Project for Elimination of Neglected Tropical Diseases (ESPEN) database (https://espen.afro.who.int/diseases/soil-transmitted-helminthiasis), where 6.2% of implementation units labelled as low STH prevalence (<20%) in 2021 recorded MDA program coverage rates >100% (range 100–285%), highlighting the inaccuracy in the denominators and numerators used. As infection levels drop, using accurate denominators and numerators is critical. The treatment coverage (the proportion of the eligible population who receive MDA) is a common evaluation metric used by control programs, with a target of at least 75% in school-age children (SAC) for STH control, as recommended by the WHO.^4^ However, few control programs examine compliance (the proportion of the eligible population who swallow the received drugs), preferably recorded by directly observed treatment (DOT). Conflating this metric with coverage thus overestimates the interpretation of MDA impact.^5^ The epidemiological measurements of coverage (including variations such as epidemiological coverage, treatment coverage, program coverage) and compliance are often miscalculated between research studies, as highlighted in two recent reviews.^5,6^ This is due to differences in denominators such as the targeted population, eligible population or estimated population and differences in numerators such as those who accepted or swallowed treatment.

Increasingly relevant to MDA program monitoring is individual longitudinal compliance, defined as individually linked compliance to repeated rounds of MDA. The importance that individual longitudinal compliance patterns have on control efforts has been demonstrated previously in studies of transmission dynamics and control impact based on individual stochastic mathematical models.^7^ In the past decade, just one field epidemiological study^8^ has reported longitudinal compliance for neglected tropical diseases (NTDs) eligible for control by preventative chemotherapy.^5^ The study examined household longitudinal compliance to four biannual rounds of MDA over 2 y for STH control in coastal Kenya.^8^ Evidence of non-random compliance to treatment was observed in adults 20–25 y of age, presenting as the age group with the highest odds of receiving partial (odds ratio [OR] 1.41 [95% confidence interval {CI} 1.22 to 1.63]) or no treatment (OR 1.81 [95% CI 1.53 to 2.14]).^8^ Studies that investigate demographic correlates with treatment behaviour are required to confirm if these reported patterns are distinct or universal. Intuitively, it would be probable that treatment-taking behaviour varies greatly due to cultural, social and personal factors. Analysing these trends against prevalence and intensity data from the same enumerated region will inform if these patterns are significant, and thus if future MDA distribution practises would benefit from targeting key demographic groups that consistently miss or refuse treatment.

Recording individual longitudinal compliance enables the identification of systematically non-treated populations, who are consistently missed and/or who persistently do not swallow medication, over multiple rounds of MDA. This population can serve as a continued source, or reservoir, of infection to other repeatedly treated individuals, as helminth parasite infection does not induce strong acquired immunity, thus individuals may be repeatedly reinfected even after drug treatment. The importance of persistently untreated or never-treated populations has been a focus of much discussion in recent research and policy formulation on the control of helminth parasites.^9^ Never-treated individuals, as well as those infrequently treated, are of importance to the overall impact of an MDA program. This is especially the case for STH and schistosomiasis (SCH), which have an estimated adult worm life expectancy in the human host of 1–2 y in high-transmission settings where reinfection is frequent. In such high-transmission settings where the basic reproductive number (R_0_) is large, a high frequency of MDA rounds (sometimes biannual) is required to greatly reduce the levels of STH infection.

The analyses presented in this article are derived from data collected by the Geshiyaro Project, which is investigating the feasibility of STH and SCH transmission interruption in endemic, low-prevalence communities in southern Ethiopia.^5^ The latest parasitological measurements in the studied area reached 6.48% (95% CI 4.56 to 8.40) for *Ascaris lumbricoides*, 3.16% (95% CI 1.80 to 4.52) of *Trichuris trichiura* and 2.84% (95% CI 1.55 to 4.13) for hookworm. Patterns of individual longitudinal compliance to treatment in populations consisting of >54 000 individuals are reported across six rounds of community-wide MDA (cMDA). The longitudinal follow-up of this large cohort, individual by individual, provides detailed data on drug-taking behaviour and the associated individual compliance patterns. The analyses described in this article focus on compliance (swallowing the received treatment) to albendazole (ALB) for the treatment of STH, since SCH infections were of very low prevalence. The patterns observed were further analysed to define their impact on cMDA-based control programs and the likelihood of transmission interruption, employing individual-based stochastic mathematical models of transmission and control impact.

## Methods

### MDA protocol

The Geshiyaro Project measures the feasibility of interrupting STH and SCH transmission by cMDA and improvements in WASH. Starting in 2018, the project will run for 7 y, completing in 2025. The project protocol and baseline findings have been published previously.^10–13^ The impact of control from different interventions deployed by the project are measured through three intervention arms: arm 1—expanded cMDA and WASH infrastructure implemented with behaviour change communication (BCC); arm 2—cMDA and the continuation of the One WASH National Program (OWNP); and arm 3—national annual school-based MDA (sMDA) and OWNP. Arm 1 and arm 2 were assigned to the districts within the Wolaita zone in the Southern Nations, Nationalities, Peoples’ Region (SNNPR) of Ethiopia. The project timeline is split into three phases: the pilot phase conducted in a single district (Bolosso Sore), the main phase (scale-up to the remaining districts in the three intervention arms) and the analysis phase. Delivery methods of cMDA were adapted during the project due to the coronavirus disease 2019 pandemic, including the replacement of fixed-point distribution employed prior to the pandemic by house-to-house distribution, avoiding mass congregation of individuals. The anthelmintics were distributed by the existing network of health extension workers (HEWs), a network of care workers stationed in each village (*kebele*) across Ethiopia underpinning the national healthcare system.^14^ Biannual rounds of cMDA were incorporated into the project in year 4, superseding annual rounds originally planned. To date, six rounds of cMDA have been delivered across 5 y of treatment to the pilot district, Bolosso Sore. This district has received more cMDA rounds than other districts due to the staggered nature of project implementation. The remaining districts have received four rounds of cMDA to date.

### Individual-level longitudinal monitoring of cMDA

A subsample of three districts in arm 1 (Bolosso Sore, Bolosso Bombe, Damot Gale) and two districts in arm 2 (Damot Weydie, Abela Abaya) were censused at baseline in 2018. All consenting participants were enrolled by biometric fingerprint (Simprints Technology, Cambridge, UK)^15^ and/or a barcoded study identification card.^10^ Both methods created a unique 11-digit identification number for individuals, assigned for the study duration, enabling registered individuals to be monitored longitudinally. This identification number also enabled linkage of the individual to their household demographics and WASH infrastructure data, both captured in the census, as well as parasitological data collected in a concurrent sentinel site cohort survey.^13^ Any individuals who were unable to be identified at each round of cMDA were newly registered to the census, including newborn babies, participants who had recently moved into the district and participants previously missed in the population census, ensuring the census remained updated for each cMDA round. All data were recorded by an HEW using the Android smartphone SurveyCTO application (Dobility, Cambridge, MA, USA). The eligible population for cMDA was calculated as the population ≥1 y of age for ALB, excluding all pregnant women. This eligible population is employed as the denominator for all cMDA coverage and compliance calculations.

### Data analysis

At cMDA, each participant was identified using their biometric fingerprint or identification card and their treatment behaviour was recorded. Each participant can therefore be categorised as either ‘missed’ or ‘contacted’ by a HEW, with the latter further categorised as ‘accepting’ or ‘refusing’ the offered drug, and subsequently ‘refusing’ or ‘swallowing’ the accepted drug. From the total population of 210 862 individuals currently registered in Bolosso Sore by the project, the longitudinal population was defined as individuals present from the baseline census to the point of analysis (present), eligible for treatment at each cMDA round—a total of 54 659 individuals. Demographic stratifications of this eligible population by age and gender were used for analysis, defining the following age groups: pre-SAC (ages 1–4y), SAC (ages 5–14 y), adolescents (ages 15–20 y), young adults (ages 21–35 y) and adults (ages 36–100 y). Analysis showing age-group categories are based on the age-group classification assigned during enrolment in year 1 (baseline).

The positive binomial and beta-binomial probability distributions were fitted to the observed frequency of cMDA rounds at which ALB was swallowed. The positive binomial distribution provides an important reference point since it describes a random pattern in compliance where the probability that an individual takes treatment at any one round of cMDA is independent of their behaviour at a previous round. The compliance data were also analysed against the beta-binomial distribution whereby each individual has an assigned independent probability of attendance for all rounds drawn from a beta-binomial distribution. The coefficient of variation (a ratio of the standard deviation divided by the mean number of cMDA rounds of treatment in which the medication was swallowed) was calculated to provide a measure of dispersion for the frequency of treatment distribution. Low values of the coefficient of variation shows a low dispersion of variation around the mean.

The Pearson’s correlation coefficient was calculated to measure the degree of randomness of individual treatment behaviour on a scale from 0, denoting entirely random, through to 1, denoting entirely systematic behaviour (either always treated or never treated). Multiple logistic regression was used to determine the impact of age group and gender on the never- and always-treated population, the output of which was interpreted by the OR, calculated from the exponentiated coefficients.

The probability of transmission interruption in Bolosso Sore was forecast employing a previously detailed individual-based stochastic model^16^ of *A. lumbricoides* transmission (the most prevalent species recorded in the Geshiyaro study area) and cMDA impact. Three different treatment compliance patterns were examined with respect to the likelihood of transmission interruption: the observed compliance (beta-binomial), random compliance (positive binomial) and completely systematic non-compliance behaviour. A fraction of the population in these simulations were never treated in each scenario (non-attendance correlation: 0.21). Within a population of 2000 people treated with ALB for *A. lumbricoides* infection, 1000 simulations were undertaken. Historical cMDA compliance of the Bolosso Sore longitudinal population was used for the implemented six rounds (1: 70.0%, 2: 65.8%, 3: 77.8%, 4: 85.1%, 5: 70.3%, 6: 67.9%), projected forward at 72.8% (average overall compliance for the six rounds) for the remaining five biannual rounds, applied uniformly across all age groups. Key epidemiological parameters were derived from the literature and baseline data, such as the baseline prevalence of *A. lumbricoides* (18%).^16^ The basic reproductive number was varied as an R_0_ of 1.7–2.9, the adult worm life expectancy at 1 y, the density-dependent parameter of worm fecundity fixed at γ=0.08, and the negative binomial aggregation parameter for adult worms was varied as k=0.06–0.18.^16^ In the simulations for systematic non-compliance, a β distribution was employed with parameters β1=0.13 and β2=0.05. Wilson (or ‘plus-4’) confidence intervals were calculated using the binconf() function from the Hmisc package in R version 4.2.2 (R Foundation for Statistical Computing, Vienna, Austria). This binomial probability interval differs from the normal, as it is asymmetric and is not affected by overshoot or zero-value intervals.

## Results

**Figure 1.**
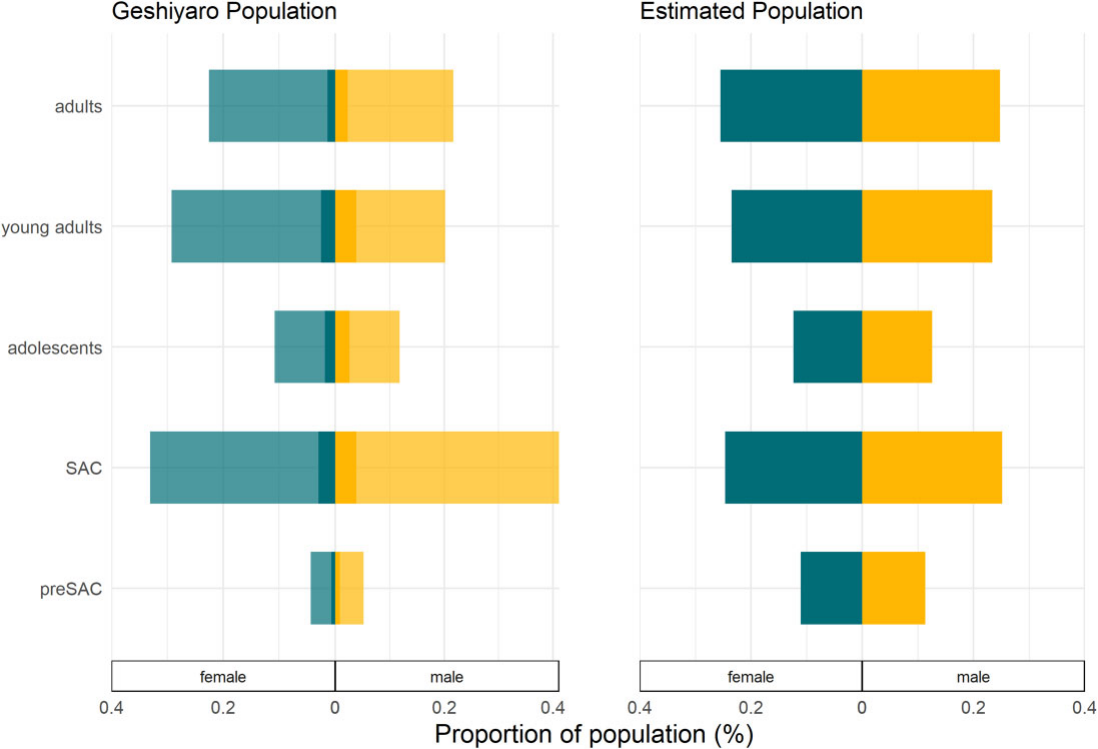
The age distribution of the Bolosso Sore population recorded by the Geshiyaro Project (n=54 659) compared with data held by the US Census Bureau for Bolosso Sore.^14^ Individuals who were able to be identified with biometrics (overall 88.7%) are shown in the paler shading. Age groupings are shown based on the age group assigned during enrolment at the baseline of the project. pre-SAC (ages 1–4 y), SAC (ages 5–14 y), adolescents (ages 15–20 y), young adults (ages 21–35 y) and adults (ages 36–100 y).

From the population registered in the district of Bolosso Sore by the Geshiyaro Project, a total of 54 659 individuals were eligible for treatment in each of the six rounds between 2018 and 2023, forming the longitudinal population for analysis. The majority of this longitudinal population was able to be biometrically identified (88.7%), as shown in Figure 1A. Most significantly, 37.9% fewer male young adults were recorded in the Geshiyaro dataset compared with female young adults (Figure 1A). The recorded age distribution of this longitudinal population by gender broadly matched the estimated data held in the US Census Bureau for Bolosso Sore^17^ (Figure 1A), with a few caveats. The pre-SAC population (female: 0.023, male: 0.025) was 79.2% and 78.1% smaller than the estimated US Census Bureau for the female and male population, respectively. Second, the Geshiyaro dataset captured fewer male young adults (59.1%) and male adults (58.5%) than that recorded by the US Census Bureau.

An average of 21 013 (range: 23 159–26 844) participants were treated in each round. ALB was swallowed by 24 172 (year 1), 23 159 (year 2), 25 415 (year 3), 26 844 (year 4a), 24 030 (year 4b) and 23 472 (year 5a) participants from this longitudinal population. Coverage (the proportion of eligible population receiving treatment) recorded across the six cMDA rounds reached 70.1% (66.1% (95% CI 65.7 to 66.5), 67.9% (95% CI 67.5 to 68.3), 70.1% (95% CI 69.7 to 70.5), 70.3% (95% CI 69.9 to 70.7), 77.8% (95% CI 77.5 to 78.1), and 85.1% (95% CI 84.8 to 85.4) from year 1 to year 5a. Compliance (the proportion of the eligible population swallowing the received drug) varied negligibly from the recorded coverage and similarly reached 65.8% (95% CI 65.4 to 66.2), 67.9% (95% CI 67.5 to 68.3%), 70.0% (95% CI 69.6 to 70.4), 70.3% (95% CI 69.9 to 70.7), 77.8% (95% CI 77.5 to 78.1), and 85.1% (95% CI 84.8 to 85.4) demonstrating a low dropout of participants who received treatment but did not swallow it.

### Frequency distributions of swallowing cMDA over six rounds

The frequency of rounds in which individuals swallowed treatment over the course of six rounds of cMDA increased from once (4.09% [n=2234]), twice (7.23% [n=3952]), three times (12.6% [n=6865]), four times (22.1% [n=12 066]), five times (29.4% [n=16 088]) and six times (24.2% [n=13 227]). Only 0.42% of the eligible population (n=227) never attended any of the six rounds of cMDA. Broken down for each age group, the pre-SAC group (OR 10.1 [95% CI 6.63 to 15.4], ref: SAC) had the highest never-treated proportion of the age groups (Table 1). Conversely, adults (OR 1.03 [95% CI 0.98 to 1.09], ref: SAC) had the highest always-treated proportion of the age groups.

**Table 1.** Mean and variance of treatment rounds stratified by age group and gender.

Age group	Gender	Population (n)	Mean	Variance	CV	PCC	Never treated, %	Never treated, OR (95% CI)	Always treated, %	Always treated, OR (95% CI)
Pre-SAC	Female	1253	3.83	2.29	0.40	0.26	1.92	10.1 (6.63 to 15.4)	12.9	0.41 (0.36 to 0.46)
	Male	1355	3.85	2.26	0.39					
SAC	Female	9523	4.51	1.72	0.29	0.49	0.19	Ref	26.5	Ref
	Male	10 615	4.50	1.83	0.30					
Adolescents	Female	3121	4.02	2.06	0.36	0.27	0.45	2.35 (1.43 to 3.80)	16.2	0.53 (0.49 to 0.57)
	Male	3056	3.91	2.25	0.38					
Young adults	Female	8412	4.47	1.93	0.31	0.42	0.55	2.85 (1.95 to 4.24)	23.8	0.85 (0.80 to 0.89)
	Male	5221	4.02	2.36	0.38					
Adults	Female	6499	4.6	1.60	0.27	0.54	0.29	1.49 (0.94 to 2.36)	27.3	1.03 (0.98 to 1.09)
	Male	5604	4.48	1.93	0.31					

Coefficient of variation is calculated for each population stratification. Pearson's correlation coefficient shown for each age bracket. ORs calculated from binary logistic regression determining the impact of the age grouping on the population that is either always or never treated, using SAC as the reference age group. Age groupings are shown using the age category assigned during enrolment at baseline.

Pre-SAC (ages 1–4 y), SAC (ages 5–14 y), adolescents (ages 15–20 y), young adults (ages 21–35 y) and adults (ages 36–100 y).

CV: coefficient of variation; PCC: Pearson's correlation coefficient.

The mean and variance of the treatment frequency distribution across six possible rounds are recorded in Table 1, stratified by age and gender. Coefficient of variation values indicate a moderate level of dispersion around the mean. No change in this pattern is detectable across stratifications by age group, gender and study population.

**Figure 2.**
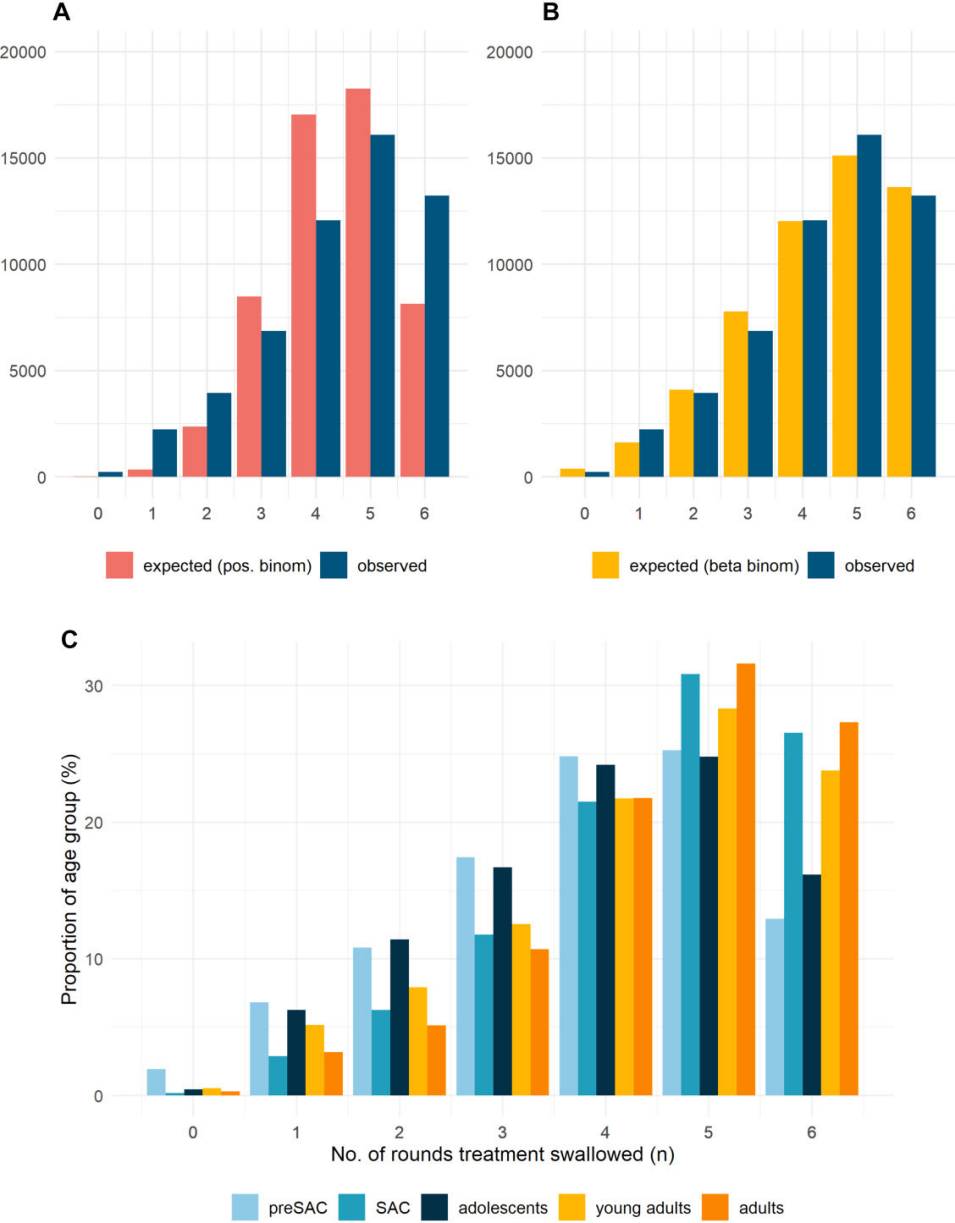
The treatment frequency recorded in Bolosso Sore (n=54 659) for six rounds of cMDA. Comparison of different probability distributions to describe compliance fitted to the observed treatment frequency data from the Bolosso Sore population enumerated by the Geshiyaro Project. Fits of the **(A)** beta-binomial compared with **(B)** the expected fit of the positive binomial (random) demonstrates that the beta-binomial distribution records the best fit to the observed compliance data. Parameters: **(A)** probability 0.27, binomial denominators 6; **(B)** μ=0.27, σ=0.15, binomial denominators 5. **(C)** The proportion breakdown of each age group into each treatment frequency category. Age groupings are shown based on the age in which the participant was when enrolled at the beginning of the project. pre-SAC (ages 1–4 y), SAC (ages 5–14 y), adolescents (ages 15–20 y), young adults (ages 21–35 y) and adults (ages 36–100 y).

The beta-binomial distribution (Figure 2A) described the data well for six cMDA rounds in Bolosso Sore (n=54 659), independent of all the stratifications by age group and gender. This fitted the observed frequency of swallowing better than the positive binomial distribution, which reported a much lower AIC value, thus demonstrating a closer goodness of fit of the beta-binomial. The departure from a random distribution pattern (Figure 2B) occurs due to excesses in the frequencies of those who were always treated across six cMDA rounds (shifting the random pattern to a more uniform one with low variance). The distribution of swallowing frequency changed between age groups, as shown in Figure 2C. Higher proportions of the pre-SAC and adolescent age groups swallowed zero to four rounds of treatment. This inverts as treatment frequency increases, whereby a higher proportion of SAC, young adults and adults swallowed five and six (all) rounds of treatment. This is consistent with the Pearson's correlation coefficient in Table 1, showing the highest probability for adults (0.54), SAC (0.49) and young adults (0.42).

### Probability of elimination

**Figure 3.**
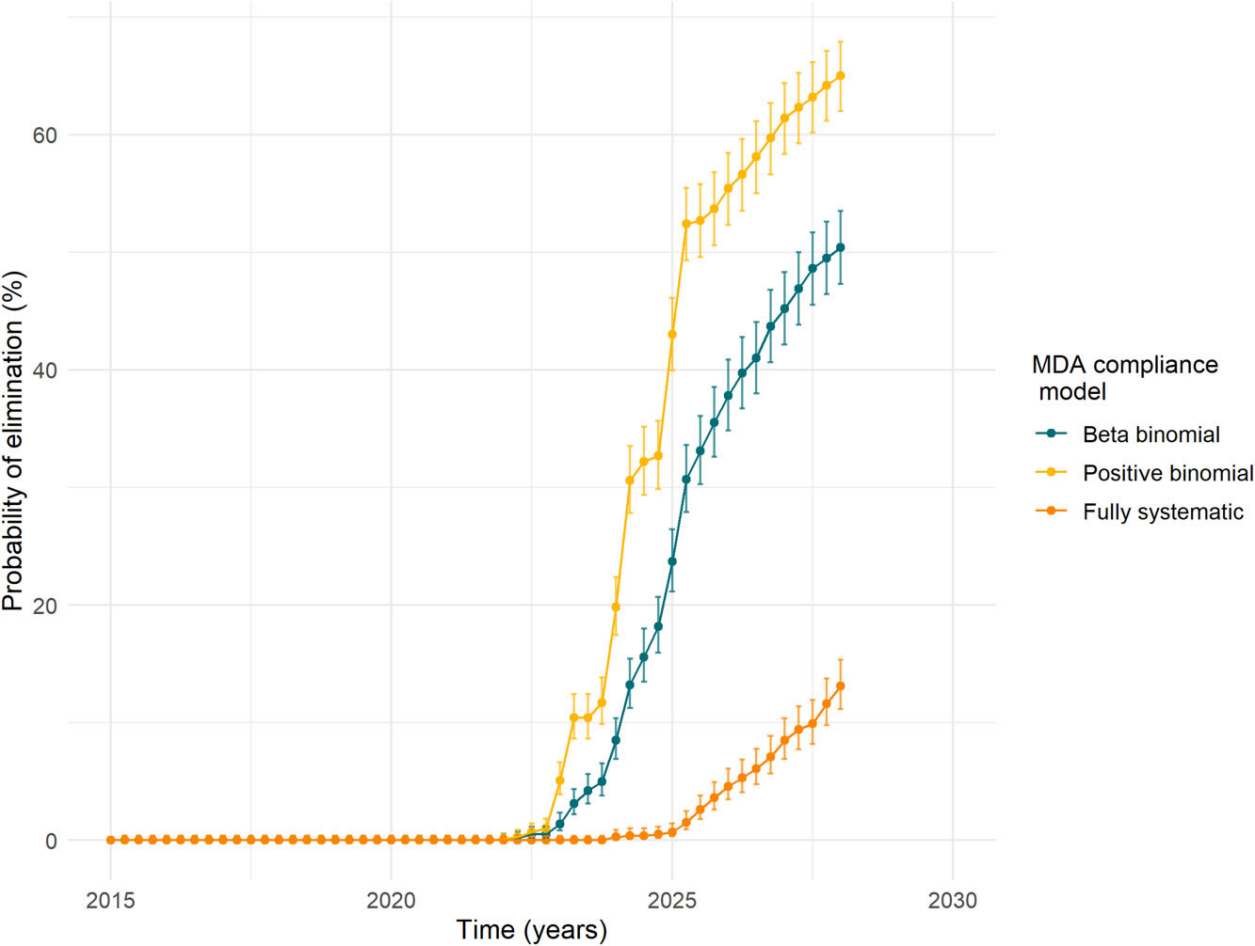
The probability of transmission interruption forecast by 1000 simulations of an individual-based, stochastic model of *A. lumbricoides* transmission in a population of 2000 people. The impact of compliance behaviour on the probability of interrupting parasite transmission is projected forward using three different population behaviour scenarios. These scenarios are the beta-binomial distribution of compliance (dark green) seen in the Geshiyaro Project, the positive binomial distribution of compliance (yellow) and a population with a simulated systematically behaving population (set proportion of 0.21 always treated) (orange). The model used the average compliance value across the six past cMDA rounds (72.8%) to project behaviour after a further five future rounds of cMDA. The starting prevalence of *A. lumbricoides* was set at 18% according to baseline data. The basic reproductive number (R_0_) was 1.7–2.9, the adult worm expectancy was 1 y, the density dependent parameter of worm fecundity was fixed at γ=0.08 and the negative binomial aggregation parameter for adult worms (k) was 0.06–0.1816. In the simulations for systematic non-compliance, a β distribution was employed with parameters β1=0.13 and β2=0.05. The asymmetric binomial probability (Wilson [or ‘plus-4’]) confidence intervals were calculated using the binconf() function from the Hmisc package in R and are shown for each probability of elimination.

Using an individual-based stochastic model of *A. lumbricoides* transmission and cMDA impact, the average probability of transmission interruption from 1000 simulated trajectories is shown in Figure 3. Over 11 cMDA rounds (the total number of rounds to be delivered by completion of the Geshiyaro Project), three cMDA treatment behaviour patterns for the treated population were examined: a beta-binomial distribution, a positive binomial distribution and a population behaving entirely systematically. Assuming cMDA compliance remains at an average 72.8% (the average compliance across all six rounds to date), the model predicts by 2028 the probability of transmission elimination is approximately 65.0% (95% CI 62.0 to 67.9) for a population following a positive binomial (random) distribution of treatment frequency, 50.4% (95% CI 47.3 to 53.5) for a population following a β binomial distribution of treatment frequency (as seen in the Geshiyaro data) and 13.1% (95% CI 11.1 to 15.3) for a population following a fully systematic fraction of non-treatment behaviour. The significant impact that compliance behaviour has upon the probability of elimination is clear.

## Discussion

This analysis of data on compliance to treatment is based on the largest dataset of individual longitudinal compliance to cMDA for STH control yet published,^5^ reporting on data describing a population of >54 000 individuals. Compliance is rarely reported accurately,^5,6^ if at all, for the NTDs eligible for MDA treatment, nor is the detailed impact of such observed compliance behaviours on helminth parasite control and possible transmission interruption. This is despite the known importance that compliance has in predicting both the epidemiological impact of MDA and the future probability of transmission interruption^18,19^ revealed by published studies of helminth transmission dynamics. Many of the NTDs can be linked to poverty and therefore are endemic in countries with a low gross domestic product. The high costs of conducting longitudinal cohort studies as part of national programming therefore limit their use in resource-poor settings. Considerable programmatic coordination and financial resources are required to first census the population to be treated and manage the subsequent data and analysis required to longitudinally link individual MDA behaviour recorded by DOT. Traditional paper-based methods currently employed by many national programs will struggle to complete this with minimal data entry errors. Accurate data acquisition requires a sophisticated infrastructure involving electronic data collection devices (mobile phones, tablets) and access to data collection software.

The highest proportion of the never-treated cohort was identified in the pre-SAC age group (infants ages 1–4 y), as well as this age group reporting the lowest probability of swallowing treatment. This may be influenced in part by difficulties in treating infants with tablets. This is concerning, as this age group is most at risk of morbidity from high *A. lumbricoides* and *T. trichiura* burdens.^20^ The lack of treatment in this vulnerable age group may consequently lead to the creation of a reservoir of infection in this age group, which can continue to seed infection. This will predominantly affect mothers and caregivers, such as older siblings, who spend prolonged time and contact with the infant age group. If these mothers are also pregnant (specifically in their third trimester), they are ineligible for treatment and thus their increased exposure to infective material will remain untreated.^4^ The proportion of participants being always treated was highest for the adult, SAC and young adult age groups. An increase in compliance with age has been similarly reported by repeated cross-sectional cohort compliance studies. The exception to this trend was the SAC age group, which recorded a very high probability of swallowing treatment. This may reflect the familiarity this age group has with national deworming programs, which routinely implement sMDA rather than cMDA. The high probability of swallowing treatment in this age group demonstrates the importance of the sensitisation of implementation activities on compliance rates. The familiarity of SAC with such deworming programs is reflected in their high compliance with treatment.

Differing compliance rates in distinct age groups will impact the prevalence of each of the STH species differently due to the differing age-dependent prevalence distributions associated with each parasite present in the study site. For example, varying compliance behaviour by age group will impact hookworm control differently from that of *A. lumbricoides* and *T. trichiura.* High hookworm infection levels occur in older adult groups compared with that of the pre-SAC and SAC groups. The reverse is true for *A. lumbricoides* and *T. trichiura.* Young adults were found to be 2.5 times more likely to be never treated compared with SAC. However, the never-treated population compromised just 0.42% of the population. Subsequent analysis of parasite prevalence with regards to stratified compliance behaviour is required to assess the significance of this phenomenon at both the individual and village level. Recent analysis has recorded evidence of a predisposition to light and heavy *A. lumbricoides* infection in the Geshiyaro cohort.^21^ Consequently, if compliance has a negligible effect on parasite prevalence, the underlying factors contributing to predisposition will require further examination.

Random (positive binomial) patterns of compliance were not observed in the data. Rather, a higher fraction of always-treated individuals than predicted by the positive binomial distribution was observed, following a beta-binomial distribution of treatment frequency. This pattern in the probability distribution of swallowing treatment over multiple rounds of cMDA was well described by the beta-binomial distribution, whereby individuals are assigned an independent probability of swallowing at all rounds, drawn from a β distribution. The non-random frequency of the ‘always-treated’ population will require further research to understand the reasons influencing this behaviour. It may be a consequence of the population being part of a large study conducted over many years, where enhanced and repeated contact with HEW implemented by the Geshiyaro Project may have induced improved compliance to treatment through high awareness of the benefits of participation. Furthermore, despite efforts to characterise the entire population of Bolosso Sore, there will be individuals missed from this dataset as noted in Figure 1 which will impact the interpretation of the low never treated proportion reported in this study.

Despite the most recent parasitological survey recording very low prevalence in Bolosso Sore, and a compliance rate just under the WHO target of 75%, the probability of elimination remained moderate, as shown in Figure 3. This highlights the fact that despite a near-zero proportion of the population never being treated, attention is required to the whole frequency distribution of drug swallowing behaviour in the targeted population. This clearly demonstrates the importance of fine-scale monitoring and evaluation of cMDA in low-prevalence settings and makes a clear case for including compliance measurements in addition to coverage in cMDA evaluation. The coverage-compliance gap has been previously noted by other authors,^22^ although not a concern for the Geshiyaro Project, presumably due to the prolonged and continued contact the project has with the community compared with a national program without a concomitant search study embedded within it. Second, individual longitudinal compliance behaviour has been shown to have a significant impact on reaching transmission break, highlighting the need for individually linked compliance to be recorded in the treated community. Understanding if population behaviour is systematic or random in nature will be crucial for the evaluation of programs in low-prevalence areas. Furthermore, such detailed datasets allow for patterns to be delineated for defined demographic groups, enabling future targeted sensitisation before cMDA to improve compliance rates. In the future, building upon this fine-scale monitoring of cMDA with global positioning system–defined infection and compliance data will allow for the impact of spatial dynamics of STH transmission and control to be explored in more detail. Critically, the next key question in cMDA monitoring and evaluation is on what spatial scale should compliance evaluation take place to assess the WHO target of elimination as a public health problem, and the subsequent goal of transmission elimination?

## Data Availability

The data underlying this article will be shared upon reasonable request to the corresponding author.
