# Corrigendum to: Patterns of individual compliance with anthelmintic treatment for soil-transmitted helminth infections in southern Ethiopia over six rounds of community-wide mass drug administration

**DOI:** 10.1093/trstmh/trad091

**Published:** 2024-04-09

This is a corrigendum to: R Maddren, B Collyer, A E Phillips, S Rayment Gomez, B Abtew, U Anjulo, D Tadele, A Sharma, A Tamiru, E Firdawek Liyew, M Chernet, R M Anderson, Patterns of individual compliance with anthelmintic treatment for soil-transmitted helminth infections in southern Ethiopia over six rounds of community-wide mass drug administration, Transactions of The Royal Society of Tropical Medicine and Hygiene, 2023; trad079, https://doi.org/10.1093/trstmh/trad079

In the originally published version of this manuscript, there was an error in author Ewnetu Firdawek Liyew's name.

This has been corrected online.

